# Rochester Epidemiology Project Data Exploration Portal

**DOI:** 10.5888/pcd15.170242

**Published:** 2018-04-12

**Authors:** Jennifer L. St. Sauver, Brandon R. Grossardt, Lila J. Finney Rutten, Veronique L. Roger, Michelle Majerus, Daniel W. Jensen, Scott M. Brue, Cynthia M. Bock-Goodner, Walter A. Rocca

**Affiliations:** 1 Division of Epidemiology, Department of Health Sciences Research, Mayo Clinic, Rochester, Minnesota; 2 Robert D. and Patricia E. Kern Center for the Science of Health Care Delivery, Mayo Clinic, Rochester, Minnesota; 3 Division of Biomedical Statistics and Informatics, Department of Health Sciences Research, Mayo Clinic, Rochester, Minnesota; 4 Department of Cardiology, Mayo Clinic, Rochester, Minnesota; 5 Health Information Management, Information Privacy Office, Olmsted Medical Center, Rochester, Minnesota; 6 Olmsted County Public Health Services, Rochester, Minnesota; 7 Department of Information Technology, Mayo Clinic, Rochester, Minnesota; 8 Department of Neurology, Mayo Clinic, Rochester, Minnesota

## Abstract

**Introduction:**

The goal of this project was to develop an interactive, web-based tool to explore patterns of prevalence and co-occurrence of diseases using data from the expanded Rochester Epidemiology Project (E-REP) medical records-linkage system.

**Methods:**

We designed the REP Data Exploration Portal (REP DEP) to include summary information for people who lived in a 27-county region of southern Minnesota and western Wisconsin on January 1, 2014 (n = 694,506; 61% of the entire population). We obtained diagnostic codes of the International Classification of Diseases, 9th edition, from the medical records-linkage system in 2009 through 2013 (5 years) and grouped them into 717 disease categories. For each condition or combination of 2 conditions (dyad), we calculated prevalence by dividing the number of persons with a specified condition (numerator) by the total number of persons in the population (denominator). We calculated observed-to-expected ratios (OERs) to test whether 2 conditions co-occur more frequently than would co-occur as a result of chance alone.

**Results:**

We launched the first version of the REP DEP in May 2017. The REP DEP can be accessed at http://rochesterproject.org/portal/. Users can select 2 conditions of interest, and the REP DEP displays the overall prevalence, age-specific prevalence, and sex-specific prevalence for each condition and dyad. Also displayed are OERs overall and by age and sex and maps of county-specific prevalence of each condition and OER.

**Conclusion:**

The REP DEP draws upon a medical records-linkage system to provide an innovative, rapid, interactive, free-of-charge method to examine the prevalence and co-occurrence of 717 diseases and conditions in a geographically defined population.

## Introduction

Changes in health information technology during the last decade and an increasing demand for data sharing and transparency have increased public access to health-related data. In particular, several web-based tools have been developed to share local, state, and national health data with audiences ranging from the general public to public health agencies and epidemiologic researchers (1–10) (Table 1). These websites and interactive tools are intended to help communities throughout the United States understand the health of their county or state and to prioritize interventions. For example, County Health Rankings and America’s Health Rankings summarize and display data on factors important to health and health management (2,5). However, the data used by these sites are collected from cross-sectional surveys of various groups of people at single points in time (eg, the Behavioral Risk Factor Surveillance System [BRFSS]) (11,12). These sites have data that can be summarized individually, by demographic characteristics, and by geographic region, but exploration of associations between different conditions or across data sets is not possible because the data are rarely linked to identifiable individuals. In addition, survey data are self-reported, and the number and type of health conditions included are limited.

**Table 1:** Comparison of Selected Interactive Web-Based Health Information Tools in the United States

Website (Reference No.)	Population Targeted	Website Purpose	Data Sources	Available Data	Explore^a^	Contact^b^
FluView (4)	All ages in the United States	Provides weekly influenza surveillance information in the United States.	State and regional laboratory reports	Influenza	No	No
Fast Stats (1)	All ages in the United States	Provides quick access to statistics on topics of public health importance.	Government sources, including National Health Interview Survey, National Hospital Ambulatory Medical Care Survey, National Ambulatory Medical Care Survey, National Survey on Drug Use and Health, state and regional laboratory reports, Healthcare Cost and Utilization Project, and National Inpatient Sample; private and global sources; others	100 topics^c^: diseases and conditions, family life, health care and insurance, risk factors, injuries, life stages, and reproductive health	No	No
County Health Rankings (2,3)	All ages in the United States	Provides county-level data on a range of factors that influence health to communities across the United States. Communities may then use the data to identify areas to focus on for interventions.	BRFSS, National Center for Health Statistics, CDC’s Diabetes Interactive Atlas, USDA Food Environment Atlas, Fatality Analysis Reporting System, others	50 topics^c^: health behaviors, clinical care, social and economic factors, physical environment	No	No
America’s Health Rankings (5)	All ages in the United States	Provides state and level data on behaviors, public and health policies, community and environmental conditions, and clinical care data.	US Department of Health and Human Services, US Department of Commerce, US Department of Education, US Department of Justice, US Department of Labor, US Environmental Protection Agency, US Census Bureau, Dartmouth Atlas of Health Care, others	68 topics^c^: health behaviors, community and environment, policy, clinical care, outcomes	No	No
CMS Data Navigator (7)	Adults aged ≥65 in the United States	Search tool for the data and information resources of CMS. Available data include data files, publications, and statistical reports.	Medicare claims data	48 topics^c^; links to summary reports and interactive tools on topics related to diseases, conditions, and health care utilization	No	No
Dartmouth Health Atlas (8,10)	Adults aged ≥65 in the United States	Uses Medicare data to provide information and summary analyses about health care markets, hospitals, and physicians across the United States.	Medicare claims data	15 topics^c^; health care cost and utilization	No	No
HCUPnet (9)	All ages in the United States	Provides an online method to query hospital inpatient, emergency department, and ambulatory care data from HCUP.	State and national inpatient databases; state ambulatory surgery and services databases; state and national emergency department databases	Health care utilization and all conditions treated in inpatient or emergency department	No	No
REP DEP	All ages in 27 counties in Midwest	Web-based tool to explore patterns of prevalence and co-occurrence of diseases using data from the Expanded REP.	Linked medical records in a geographically defined population	717 conditions	Yes	Yes

Abbreviations: AHRQ, Agency for Healthcare Research and Quality; BRFSS, Behavioral Risk Factor Surveillance System; CDC, Centers for Disease Control and Prevention; CMS, Centers for Medicare & Medicaid Services; HCUP, Healthcare Cost and Utilization Project; REP DEP, Rochester Epidemiology Project Data Exploration Portal; USDA, US Department of Agriculture.

a Is it possible to explore the co-occurrence of 2 diseases or conditions among people in the county?

b Can investigators contact people with a given disease or condition to invite them to participate in an observational study or a clinical trial?

c “Topics” refers to health-related, social, environmental, or economic areas of public health importance.

Other interfaces are available from the Centers for Medicare & Medicaid Services (CMS Data Navigator) (7), the Dartmouth Atlas of Health Care (8), and the Agency for Healthcare Research and Quality’s Healthcare Cost and Utilization Project website (HCUPnet) (9). These websites allow users to summarize Medicare and Medicaid claims information (7,8) as well as information from state and national inpatient and emergency department databases (13). The CMS Data Navigator provides access to published reports on various topics (7), and users can explore the prevalence and co-occurrence of 20 chronic conditions (through the Medicare Chronic Conditions Dashboard) (14,15). The Dartmouth Atlas website tools also aggregate and summarize Medicare data but allow for more customized queries focused on health care utilization and outcomes (8). However, these sites provide limited information on specific diseases and conditions, particularly those that are rare. In addition, Medicare predominantly serves the population aged 65 years or older. Therefore, these sites are of limited use for understanding the health of younger populations.

Finally, the HCUPnet website allows users to explore detailed data available from State and National Inpatient Databases, the State Ambulatory Surgery and Services Database, and State and National Emergency Department Databases (9). Information is available across all ages and both sexes, and on all conditions that occur during an inpatient or emergency department visit. However, these data sets lack information on outpatient visits, and the interactive tools do not allow users to explore associations across conditions.

Our objective was to develop an interactive, web-based tool, the Rochester Epidemiology Project (REP) Data Exploration Portal (DEP), to display data on the prevalence and co-occurrence of 717 conditions from the expanded REP medical records-linkage system, which collects data from participating health care providers in in a 27-county region of southern Minnesota and western Wisconsin (16–18).

## Methods

Development of the REP DEP took place from May 2016 through May 2017. The Mayo Clinic and Olmsted Medical Center institutional review boards approved this project. We designed the tool to allow users to access summary information on the health conditions of people in a 27-county region of southern Minnesota and western Wisconsin. First, we coded information on 717 conditions. We then calculated 1) the prevalence of each selected condition, 2) the prevalence of combinations of 2 conditions (dyads), and 3) observed-to-expected ratios (OERs) to measure the excess co-occurrence of dyads. 

### Data source

From 1966 through 2010, the REP focused on the health of the Olmsted County, Minnesota, population (16,17). In 2010, the REP expanded to encompass people living in a 27-county region of southern Minnesota and western Wisconsin (18). The expanded REP (E-REP) captures data on all health conditions that come to medical attention at the participating health care providers in this region. The data are electronically available at the person-level for community members of all ages and are collected from all health care providers in the REP system, from inpatient records, outpatient records, and emergency departments (17,18).

We used REP DEP summary data for all people who lived in this region on January 1, 2014, and who were identified by using the E-REP infrastructure (18,19). The REP DEP includes data for nearly 700,000 persons (61% of the entire population in the region) (Table 2). Characteristics of the REP DEP population are similar to those of the entire 27-county region and of Minnesota and Wisconsin (Table 3). The age and sex distribution is also largely similar to that of the entire US population (20); however, people living in the 27 counties, compared with the entire US population, have a higher level of education and are less likely to be of a nonwhite race or Hispanic ethnicity (Table 3).

**Table 2:** Rochester Epidemiology Project Census Population Included in the Data Exploration Portal on January 1, 2014^a^

County	By Age Group, y					
	0–20	21–39	40–64	65–79	≥80	All Ages
**Men**						
**Minnesota**						
Olmsted	21,181	19,112	22,255	6,733	2,231	71,512
Dodge	2,807	2,014	2,995	838	272	8,926
Mower	5,273	4,744	5,567	1,865	900	18,349
Goodhue	3,511	3,580	5,893	2,275	832	16,091
Fillmore	1,857	1,482	2,439	1,005	373	7,156
Wabasha	2,332	1,963	3,138	1,226	412	9,071
Winona	1,679	1,542	2,293	979	284	6,777
Houston	981	963	1,382	545	197	4,068
Freeborn	3,268	3,051	4,584	1,831	791	13,525
Steele	4,435	3,541	4,864	1,515	569	14,924
Rice	1,925	1,591	2,535	1,167	432	7,650
Blue Earth	3,934	4,400	4,424	1,631	712	15,101
Waseca	2,110	1,718	2,431	873	343	7,475
Faribault	1,141	996	1,620	715	351	4,823
Martin	1,819	1,474	2,364	979	488	7,124
Watonwan	1,008	774	1,076	431	234	3,523
Brown	509	510	854	463	253	2,589
Nicollet	1,882	1,812	2,451	963	394	7,502
Le Sueur	1,601	1,361	2,288	933	337	6,520
**Wisconsin**						
Eau Claire	6,457	7,753	8,810	3,113	1,124	27,257
Trempealeau	2,201	1,829	2,761	868	297	7,956
La Crosse	6,342	7,985	8,359	2,315	657	25,658
Buffalo	925	824	1,367	548	202	3,866
Pepin	509	425	660	328	130	2,052
Dunn	4,352	4,070	5,061	1,787	522	15,792
Barron	2,139	2,431	3,234	1,365	429	9,598
Chippewa	2,827	2,905	4,461	1,656	507	12,356
**All counties**	**89,005**	**84,850**	**110,166**	**38,947**	**14,273**	**337,241**

**Women**						
**Minnesota**						
Olmsted	20,594	22,143	24,311	7,942	3,511	78,501
Dodge	2,690	2,175	2,968	901	441	9,175
Mower	5,014	4,958	5,419	2,136	1,510	19,037
Goodhue	3,314	3,906	5,978	2,436	1,358	16,992
Fillmore	1,764	1,665	2,472	1,063	577	7,541
Wabasha	2,310	2,048	3,171	1,350	576	9,455
Winona	1,512	1,765	2,411	957	402	7,047
Houston	961	904	1,368	581	316	4,130
Freeborn	3,110	3,225	4,554	2,045	1,199	14,133
Steele	4,314	3,724	4,795	1,698	976	15,507
Rice	1,967	2,256	3,031	1,272	554	9,080
Blue Earth	3,803	5,034	4,606	1,793	1,158	16,394
Waseca	1,971	1,941	2,714	900	523	8,049
Faribault	1,015	1,079	1,678	876	530	5,178
Martin	1,756	1,645	2,442	1,085	827	7,755
Watonwan	985	863	1,078	487	357	3,770
Brown	457	609	909	500	346	2,821
Nicollet	1,839	2,324	2,518	1,073	589	8,343
Le Sueur	1,558	1,667	2,401	1,030	517	7,173
**Wisconsin**						
Eau Claire	6,338	8,017	8,977	3,539	1,976	28,847
Trempealeau	2,103	1,879	2,613	960	490	8,045
La Crosse	6,203	7,565	8,433	2,590	1,355	26,146
Buffalo	943	812	1,312	537	267	3,871
Pepin	428	450	756	323	151	2,108
Dunn	4,135	4,096	5,131	1,912	900	16,174
Barron	2,114	2,188	3,067	1,406	644	9,419
Chippewa	2,652	3,024	4,338	1,797	763	12,574
**All counties**	**85,850**	**91,962**	**113,451**	**43,189**	**22,813**	**357,265**

**Both Sexes**						
**Minnesota**						
Olmsted	41,775	41,255	46,566	14,675	5,742	150,013
Dodge	5,497	4,189	5,963	1,739	713	18,101
Mower	10,287	9,702	10,986	4,001	2,410	37,386
Goodhue	6,825	7,486	11,871	4,711	2,190	33,083
Fillmore	3,621	3,147	4,911	2,068	950	14,697
Wabasha	4,642	4,011	6,309	2,576	988	18,526
Winona	3,191	3,307	4,704	1,936	686	13,824
Houston	1,942	1,867	2,750	1,126	513	8,198
Freeborn	6,378	6,276	9,138	3,876	1,990	27,658
Steele	8,749	7,265	9,659	3,213	1,545	30,431
Rice	3,892	3,847	5,566	2,439	986	16,730
Blue Earth	7,737	9,434	9,030	3,424	1,870	31,495
Waseca	4,081	3,659	5,145	1,773	866	15,524
Faribault	2,156	2,075	3,298	1,591	881	10,001
Martin	3,575	3,119	4,806	2,064	1,315	14,879
Watonwan	1,993	1,637	2,154	918	591	7,293
Brown	966	1,119	1,763	963	599	5,410
Nicollet	3,721	4,136	4,969	2,036	983	15,845
Le Sueur	3,159	3,028	4,689	1,963	854	13,693
**Wisconsin**						
Eau Claire	12,795	15,770	17,787	6,652	3,100	56,104
Trempealeau	4,304	3,708	5,374	1,828	787	16,001
La Crosse	12,545	15,550	16,792	4,905	2,012	51,804
Buffalo	1,868	1,636	2,679	1,085	469	7,737
Pepin	937	875	1,416	651	281	4,160
Dunn	8,487	8,166	10,192	3,699	1,422	31,966
Barron	4,253	4,619	6,301	2,771	1,073	19,017
Chippewa	5,479	5,929	8,799	3,453	1,270	24,930
**All counties**	**174,855**	**176,812**	**223,617**	**82,136**	**37,086**	**694,506**

a Table includes data only for persons who gave permission for all or part of their medical record information to be used for research purposes. The complete population enumerated by the Rochester Epidemiology Project Census on January 1, 2014 comprised 763,695 persons (369,403 men and 394,292 women); therefore, the participation rates were 90.9% overall, 91.3% for men, and 90.6% for women.

**Table 3:** Demographic, Racial/Ethnic, and Socioeconomic Characteristics of the 27-County Region in the Rochester Epidemiology Project Data Exploration Portal (REP-DEP), Minnesota and Wisconsin, and the Entire US Population in 2014

Characteristics	27-County Region, REP DEP	27-County Region, US Census^a^	Minnesota and Wisconsin, US Census^a^	US Total, US Census^a^
**Total population**	694,506	1,139,548	11,216,557	318,907,401
**Demographic**				
Aged ≥18 y, %	78.8	77.8	77.0	76.9
Aged ≥65 y, %	17.2	16.0	14.8	14.5
Median age, y	39.4	38.2	38.5	37.7
Men, %	48.6	49.8	49.7	49.2
**Racial/ethnic, %**				
White	87.6	93.3	86.8	77.3
Nonwhite	8.2	6.7	13.2	22.7
Black	2.8	2.2	6.2	13.2
Asian	2.0	2.6	3.7	5.5
American Indian/Alaska Native	0.2	0.5	1.2	1.2
Native Hawaiian/Pacific Islander	0.1	0.1	0.1	0.2
Other and mixed^b^	3.0	1.5	2.0	2.5
Unknown race	4.2	—	—	—
Hispanic or Latino	4.6	4.2	5.8	17.4
**Socioeconomic characteristics, %**				
≥High school diploma	93.5^c^	92.1	91.7	86.7
≥Bachelor’s degree	34.1^c^	26.7	30.7	29.7
Persons below federal poverty level	—	11.2	11.2	14.7

a The estimates for 2014 are from the US Census (20).

b Other and mixed race includes persons who reported their race as “other” or “mixed” in the Expanded Rochester Epidemiology Project and persons who specified “Two or more races” in the US Census.

c Data on education were available for 46.1% of the Expanded REP DEP population aged ≥25 years.

The REP DEP includes information only for persons who have given permission for their medical records to be used for research purposes (91% of the sample population) (18). All information is available in aggregate summary form only, and the REP DEP reports values only when an age, sex, and/or county stratum contains 11 or more people. 

### Medical conditions

We developed the REP DEP to offer information on 717 conditions. We obtained diagnosis codes of the International Classification of Diseases, 9th edition (ICD-9) from patient medical records between January 1, 2009 and December 31, 2013, and we grouped codes by using 2 coding systems. First, we grouped ICD-9 codes into categories defined by the Agency for Healthcare Research and Quality as part of the Hospital Cost and Utilization Project (21). We used the Clinical Classification Software (CCS) to define a total of 690 conditions: 283 main-level, 376 sub-level, and 31 sub-sub-level code groupings (22,23). Second, we created 20 additional groupings by using diagnosis code categories defined by the US Department of Health and Human Services for studying multiple chronic conditions (24); we also added anxiety disorders to this list for a total of 21 chronic condition groupings.

Finally, we identified a series of 6 mental and neurological conditions that were well characterized by a single ICD-9 code, and we created a REP-defined sublevel grouping (Alzheimer’s disease; dementia with Lewy bodies; Huntington’s chorea; restless legs syndrome; amyotrophic lateral sclerosis; and mild cognitive impairment). The complete list of ICD-9 codes defining each of the 717 conditions is available on the REP DEP website: http://rochesterproject.org/portal/.

### Prevalence

We calculated the prevalence of each condition and the prevalence of dyads. A person was determined to have a condition if the medical record showed one or more diagnostic codes from the corresponding code grouping in the 5-year period before January 1, 2014. For each condition, we calculated prevalence by dividing the number of people with a specified condition (numerator) by the total number of people in the population (denominator). We calculated prevalence overall for a single condition and for dyads, and in strata by age, sex, and county.

### Observed-to-expected ratios (OERs)

We calculated observed-to-expected ratios (OERs) to measure the excess co-occurrence of dyads (25,26). We divided the number of observed people with 2 conditions by the expected number of people with both conditions under the assumption of conditional independence. We computed the expected numbers of people at the single-year-of-age level. For example, the expected number of people with both conditions for the age stratum 0 to 20 years was calculated for single years of age from 0 to 20 and then summed. An OER of less than 1.0 indicates that fewer people were observed with co-occurring conditions than would be expected under the assumption of conditional independence. An OER greater than 1.0 indicates that more people with co-occurring conditions were observed than would be expected under the assumption of conditional independence.

We determined whether the OER differed significantly from 1.0 by calculating 95% confidence intervals directly from the Poisson distribution using Daly’s method (27). We used ColorBrewer version 2.0 to illustrate the range of OERs in color (28). Prevalence and OER values for each county were directly standardized by age and sex to the total 2010 US Decennial Census population (Appendix) to facilitate comparison across counties while accounting for differences in age and sex distributions (20).

## Results

The first version of the REP DEP was launched in May 2017 and can be accessed at http://rochesterproject.org/portal/. To search for a condition, users can click on the box “Characteristic A selection” and start typing the text of the condition of interest. The selection list will narrow to include conditions matching the typed text. The second condition, Characteristic B, is selected in the same way. Users can display results by using the “Prevalence” tab and the “Geography” tab.

### Prevalence tab

**Figure 1:**
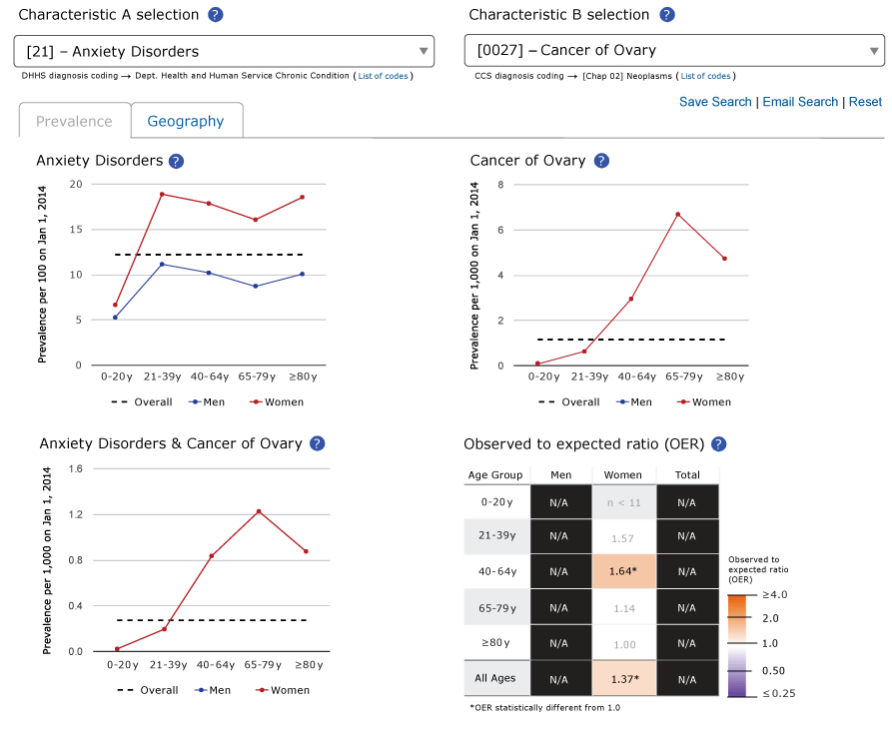
Screenshot of the “Prevalence” tab for anxiety disorders, cancer of the ovary, and the dyad consisting of anxiety disorders and cancer of the ovary in the Rochester Epidemiology Project Data Exploration Portal.

The prevalence tab for 2 selected conditions shows the prevalence of each condition as a line graph, by sex and overall, across 5 age groups (Figure 1). The tab also shows a graph of the prevalence of the 2 conditions co-occurring, by sex and overall, across 5 age groups. In addition, the tab shows a table of OERs by sex and age group. OERs are not calculated if fewer than 11 persons with both conditions are observed in a group. Similarly, for conditions affecting only one sex (eg, cancer of ovary), “NA [not applicable]” is reported in the table of OERs in the column for the unaffected sex and in the column for both sexes (“Total”). OER values that are significantly different from 1.0 are shaded with purple (OER < 1.0) and orange (OER > 1.0). OER values are not shaded if the OER is not significantly different from 1.0. For example, ovarian cancer and anxiety disorders can never co-occur in men, but they do co-occur more frequently than expected in women aged 40 to 64 years (Figure 1).

### Geography tab

**Figure 2:**
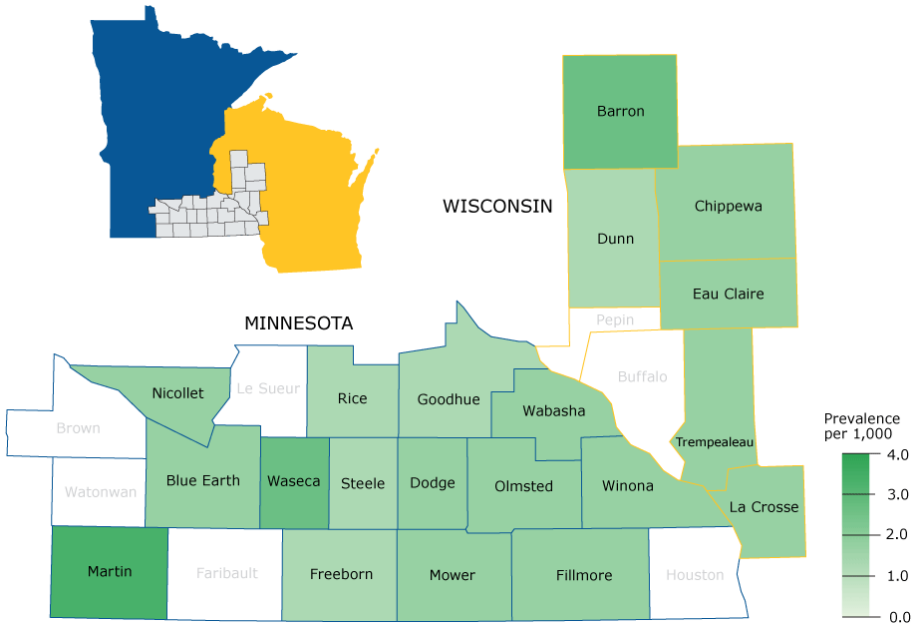
Screenshot of the “Geography” tab displaying the prevalence of cancer of the ovary (per 1,000 women) across the 27-county region in the Rochester Epidemiology Project Data Exploration Portal.

Users can also display the prevalence or OER for a selected condition by county and by sex (Figure 2). The standardized prevalence and OERs are displayed in a pop-up box when the cursor hovers over a selected county. The map in the sample screenshot indicates that the age-standardized prevalence of ovarian cancer varies across the 27-county region and is highest in Martin County.

## Discussion

We developed the interactive, web-based REP DEP to display the prevalence and co-occurrence of 717 diseases and conditions recorded in the E-REP records-linkage system. We expect the REP DEP to be useful to local residents, health care practitioners, and local administrators in understanding patterns of disease in this Midwestern region. The data may also serve as a benchmark for other communities and may provide a cost-effective way for researchers to explore whether an association between 2 conditions exists before conducting a full epidemiologic study.

The REP DEP includes data on all conditions that come to medical attention, regardless of whether the care was delivered in the outpatient, inpatient, or emergency department setting. As such, it overcomes limitations of other websites that include only a limited number of conditions or only data from inpatient or emergency department settings (2–6,13), and it allows users to obtain prevalence estimates on both common and rare conditions and to include both inpatient and outpatient diagnoses. Second, the REP DEP includes data for all age groups, overcoming the limitations of websites that rely predominantly on Medicare data (7,8). We expect REP DEP prevalence estimates to be particularly useful for public health and care delivery organizations in this 27-county region in ranking their most urgent community health priorities. For example, tax-exempt hospitals must conduct a community health needs assessment every 3 years in compliance with the Patient Protection and Affordable Care Act, and they must develop a community health improvement plan to address the most urgent priorities (29). The REP DEP can identify the prevalence of a wide array of medical conditions, and, in the future, will provide a way to determine whether the prevalence of key conditions changes over time.

REP DEP data are also linked at the person-level, which allows users to explore associations between conditions. This type of data exploration is not possible on other websites that aggregate de-identified data from different sources and populations (2–6). In addition, the underlying data included in the REP DEP are linked to patient identifiers through the E-REP research infrastructure (18). With appropriate approvals, the E-REP can be leveraged for recruiting study participants, and these participants may be followed via their linked medical records to cost-effectively assess outcomes that come to medical attention. Therefore, the REP DEP offers a method for determining whether a given community includes a sufficient number of potential participants for a community-based clinical trial (30).

This study has limitations. Data are available for 61% of the population residing in the 27-county region. Participants may differ from nonparticipants, and prevalence estimates may be biased. Conditions that are diagnosed and treated at health care providers that do not participate in the E-REP may be missed, and the true prevalence of some conditions may be underestimated. The age and sex distribution of the population included in the REP DEP is similar to US Census estimates for the 27-county region, but participants may differ from nonparticipants in other factors that influence health (eg, socioeconomic status).

Second, we informally compared REP DEP prevalences for 5 common chronic conditions with 2015 prevalence estimates for the state of Minnesota from the BRFSS (31); however, we did not perform formal statistical testing for the differences. Prevalence estimates were similar for asthma (REP DEP, 8% vs BRFSS, 7%) and depression (REP DEP, 14% vs BRFSS, 19%). However, REP DEP estimates were higher for diabetes (REP DEP, 14% vs BRFSS, 8%), and lower for arthritis (REP DEP, 15% vs BRFSS, 22%) and hyperlipidemia (REP DEP, 24% vs BRFSS, 32%). These discrepancies highlight the fact that different data collection methods are likely to yield different prevalence estimates. Methodologic differences between the BRFSS and the REP DEP preclude a more formal comparison. The BRFSS estimates were obtained from adult participants reporting whether they had ever been told that they had the condition of interest. By contrast, the REP DEP prevalence estimates were obtained from data on participants of all ages whose medical record had at least one ICD-9 code of interest in a 5-year time frame. The underlying ICD-9 codes were obtained from billing data and were not validated. Therefore, the prevalences and OERs generated by the REP-DEP may deviate from the truth. This limitation is common to all publicly accessible databases. In addition, the sensitivity and specificity of a single ICD-9 code for a condition of interest varies (32,33). Therefore, further validation studies may be necessary, depending on the condition of interest. Finally, the BRFSS estimates were for the adult population of the entire state of Minnesota, whereas the REP DEP estimates were for persons of all ages residing in a region that includes southern Minnesota and western Wisconsin. Inclusion of children in the estimates will underestimate the prevalence of chronic diseases that predominantly affect adults. However, variability in prevalence estimates may also reflect true prevalence differences between the REP DEP and BRFSS populations.

Third, ICD-9 codes were grouped into larger categories. Specific diagnoses may have been overly aggregated, resulting in the inability to test for associations of interest. For example, the diagnostic codes for Alzheimer’s disease are part of the larger category of “delirium, dementia, and amnestic and other cognitive disorders.” However, Alzheimer’s disease is a major research focus for many investigators; therefore, we included Alzheimer’s disease as an option in our search tool. In the second release of the REP DEP (January 2018), we included a series of more specific conditions. Finally, once we have 3 full years of data accumulation (2014–2016), we will add trend graphs to explore increases or decreases in the prevalence of conditions across calendar years.

The REP DEP covers a geographically defined Midwestern population, and the prevalence of medical conditions will be different in other United States communities, depending on the characteristics of the underlying population. However, the REP DEP data may still serve as a useful benchmark for other communities. In particular, it is often difficult to obtain baseline prevalence data for rare conditions. The REP DEP provides prevalence estimates for all conditions in this population, and it offers a free, rapid way to obtain comparison data. The REP DEP also provides an example of how other communities might leverage and display their own data to inform local planning efforts.

Finally, the underlying biological processes that lead to the development and co-occurrence of diseases and conditions are less likely to vary from community to community. Therefore, the OERs that can be obtained through the REP DEP are likely generalizable to other populations. As such, these data provide an avenue for researchers to determine whether 2 conditions are associated before conducting a larger, resource-intensive epidemiologic study.

The REP DEP provides a rapid, interactive, free-of-charge method to examine the prevalence and co-occurrence of 717 diseases and conditions in a large, Midwestern population. The REP DEP will be useful to local communities for understanding the prevalence of virtually all conditions in this region. In addition, these data may serve as a benchmark for other communities, particularly for rare conditions. The REP DEP can provide preliminary data for investigators who are considering further studies of the co-occurrence of diseases or are assessing the feasibility of a community-based clinical trial.

In January 2018, we released a new version of the REP DEP. This updated version of the portal allows users to choose from among 1,376 characteristics, including diagnosis-based medical conditions, procedures and surgeries, prescription medications, and demographic characteristics (eg, race, ethnicity, smoking status, overweight and obesity categories). In addition, users may now choose to define a characteristic as occurring in either the 5-year period before prevalence date or in a 1-year period before prevalence date. These updates to the REP DEP give users more flexibility to explore the relationships between characteristics. Complete details can be found in the updated REP DEP User Manual on the portal Documentation tab (http://www.rochesterproject.org/portal/).

## Appendix Total 2010 US Census Population Used for Direct Standardization

**Table:**

Age, y^a^	Population, N		
	Men	Women	Total
0–1	2,014,276	1,929,877	3,944,153
1–2	2,030,853	1,947,217	3,978,070
2–3	2,092,198	2,004,731	4,096,929
3–4	2,104,550	2,014,490	4,119,040
4–5	2,077,550	1,985,620	4,063,170
5–6	2,072,094	1,984,764	4,056,858
6–7	2,075,319	1,991,062	4,066,381
7–8	2,057,076	1,973,503	4,030,579
8–9	2,065,453	1,981,033	4,046,486
9–10	2,119,696	2,028,657	4,148,353
10–11	2,135,996	2,036,545	4,172,541
11–12	2,103,264	2,011,151	4,114,415
12–13	2,100,145	2,006,098	4,106,243
13–14	2,104,914	2,013,099	4,118,013
14–15	2,135,543	2,030,439	4,165,982
15–16	2,177,022	2,065,798	4,242,820
16–17	2,216,034	2,100,105	4,316,139
17–18	2,263,153	2,132,142	4,395,295
18–19	2,305,473	2,195,382	4,500,855
19–20	2,341,984	2,243,250	4,585,234
20–21	2,308,319	2,210,810	4,519,129
21–22	2,223,198	2,131,096	4,354,294
22–23	2,177,797	2,086,845	4,264,642
23–24	2,140,799	2,057,772	4,198,571
24–25	2,164,063	2,085,300	4,249,363
25–26	2,161,308	2,101,042	4,262,350
26–27	2,097,088	2,055,217	4,152,305
27–28	2,140,651	2,108,218	4,248,869
28–29	2,118,605	2,096,644	4,215,249
29–30	2,117,939	2,105,137	4,223,076
30–31	2,160,802	2,124,866	4,285,668
31–32	1,988,155	1,982,063	3,970,218
32–33	1,994,476	1,992,371	3,986,847
33–34	1,936,863	1,943,287	3,880,150
34–35	1,916,204	1,923,012	3,839,216
35–36	1,980,916	1,975,518	3,956,434
36–37	1,890,595	1,911,492	3,802,087
37–38	1,953,386	1,981,059	3,934,445
38–39	2,049,720	2,072,160	4,121,880
39–40	2,167,405	2,197,391	4,364,796
40–41	2,191,249	2,192,025	4,383,274
41–42	2,047,818	2,067,167	4,114,985
42–43	2,028,653	2,047,451	4,076,104
43–44	2,035,990	2,069,115	4,105,105
44–45	2,090,267	2,121,229	4,211,496
45–46	2,237,450	2,271,418	4,508,868
46–47	2,230,982	2,288,779	4,519,761
47–48	2,238,248	2,297,017	4,535,265
48–49	2,237,734	2,301,062	4,538,796
49–50	2,264,671	2,341,230	4,605,901
50–51	2,300,354	2,359,941	4,660,295
51–52	2,190,766	2,273,865	4,464,631
52–53	2,207,246	2,293,600	4,500,846
53–54	2,141,354	2,239,000	4,380,354
54–55	2,093,554	2,198,445	4,291,999
55–56	2,073,473	2,181,236	4,254,709
56–57	1,956,141	2,081,372	4,037,513
57–58	1,905,355	2,031,031	3,936,386
58–59	1,834,808	1,960,120	3,794,928
59–60	1,753,871	1,887,398	3,641,269
60–61	1,745,507	1,875,624	3,621,131
61–62	1,679,077	1,813,519	3,492,596
62–63	1,712,692	1,850,490	3,563,182
63–64	1,672,329	1,811,555	3,483,884
64–65	1,267,895	1,389,236	2,657,131
65–66	1,273,310	1,407,451	2,680,761
66–67	1,248,276	1,390,865	2,639,141
67–68	1,248,906	1,400,459	2,649,365
68–69	1,087,296	1,236,376	2,323,672
69–70	994,759	1,147,565	2,142,324
70–71	945,611	1,097,510	2,043,121
71–72	900,148	1,049,175	1,949,323
72–73	853,726	1,010,549	1,864,275
73–74	787,863	949,097	1,736,960
74–75	756,624	927,863	1,684,487
75–76	721,008	899,069	1,620,077
76–77	647,804	823,266	1,471,070
77–78	631,884	823,446	1,455,330
78–79	602,458	797,665	1,400,123
79–80	579,234	791,961	1,371,195
80–81	543,559	764,952	1,308,511
81–82	494,870	717,995	1,212,865
82–83	462,983	698,438	1,161,421
83–84	419,831	654,978	1,074,809
84–85	373,131	612,590	985,721
85–86	336,819	577,904	914,723
86–87	293,120	521,091	814,211
87–88	249,803	463,105	712,908
88–89	217,436	423,183	640,619
89–90	176,689	361,309	537,998
90–91	136,948	298,615	435,563
91–92	103,799	241,188	344,987
92–93	81,072	200,317	281,389
93–94	59,037	157,941	216,978
94–95	43,531	125,918	169,449
95–96	30,951	98,766	129,717
96–97	21,424	73,799	95,223
97–98	14,556	53,582	68,138
98–99	9,259	36,641	45,900
≥99	15,235	70,395	85,630
**All ages**	**151,781,326**	**156,964,212**	**308,745,538**

^a^ Age intervals include the lower value and exclude the upper value. For example, the interval 0–1 includes all persons of age birth through the day before the first birthday.
